# The behaviour of metals in deep fluids of NE Iceland

**DOI:** 10.1038/s41598-022-26028-x

**Published:** 2022-12-19

**Authors:** Marion Saby, Vincent van Hinsberg, Daniele L. Pinti, Kim Berlo, Bjarni Gautason, Ásgerður Sigurðardóttir, Kevin Brown, Océane Rocher

**Affiliations:** 1 GEOTOP and Département des Sciences de la Terre et de l’atmosphère, Université du Québec à Montréal, Montréal, QC H3C 3P8 Canada; 2 GEOTOP and Department of Earth and Planetary Sciences, McGill University, 34, University Street, Montréal, Canada; 3 ÍSOR Iceland GeoSurvey, Orkugardur, Grensásvegur 9, Reykjavik, Iceland; 4 Landsvirkjun, Háaleitisbraut 68, Reykjavik, Iceland; 5 Geokem, P.O. Box 30-125, Barrington, Christchurch, 8244 New Zealand; 6 GeoRessources - UMR 7359, Université de Lorraine, Vandoeuvre-Les-Nancy, France

**Keywords:** Geochemistry, Volcanology

## Abstract

In this contribution, we present some of the first data on the elemental signature of deep crustal fluids in a basalt-hosted, low-chloride magmatic-hydrothermal system. Down-hole fluid samples (850–1600 m) from wells in the Theistareykir and Krafla geothermal fields in the Northern Volcanic Zone of Iceland were combined with well-head samples of condensed vapor, cuttings of altered rock, and fresh basalt (being some of the first concentration data for volatile and semi-volatile elements (Sb, Tl, Bi, Cd and As) for this area of Iceland). Results show that the deep fluids are relatively enriched in base metals and (semi)-volatile metals (in particular Te, Hg, Re and Tl) compared to local basalt. We interpret this enrichment in volatile metals to reflect a significant element input from magma degassing. Boiling of this deep fluid results in a well-head fluid composition that is significantly depleted in most elements. This well-head fluid has a distinct elemental signature, including a depletion in Sb that is mirrored in the altered rocks, and a depletion in the base metals that shows their selective sequestration in scale minerals, likely sulphides. As expected, the element content and patterns in surface fluids can thus not be interpreted to directly reflect that of the deep reservoir fluid. The behaviour of elements in Theistareykir and Krafla fluids is consistent, and largely agrees with similar data obtained for the Reykjanes geothermal system in SW Iceland. We therefore posit that our results are representative for this geological setting and indicate a significant magmatic degassing cation input to deep fluids, variably modified by water–rock interaction.

## Introduction

Hydrothermal fluids are increasingly of interest as a sustainable and clean source of heat and energy and are regarded as one of the cornerstones of transitioning to a CO_2_ neutral world^1^. Iceland is at the forefront of this development, and currently obtains 62% of its energy needs from geothermal plants^2^. Sustainable exploitation does require an in-depth understanding of the fluid sources and evolution in these systems and investigating geothermal fluid composition is therefore essential^3–5^.

Geothermal resources also provide a unique opportunity to access deep fluids in production wells and study the mobility of elements in the crust. Active hydrothermal systems related to magma intrusions (i.e., magmatic-hydrothermal systems) are historically regarded as analogues to ore-forming systems for many decades^1,6–12^ and can therefore elucidate key questions in economic geology including the sources and fluxes of metals and fluids, and the aqueous concentrations and speciation of ore elements^10,13–15^. In particular, the elemental and isotopic composition of geothermal fluids can put constraints on the respective contributions of magma degassing and rock leaching to the metal content of active magmatic-hydrothermal systems^9,16^.

Fluids in geothermal fields are normally sampled at the wellhead, given the technical challenges of sampling downhole fluids. The compositions of these surface fluids are commonly regarded as representative of deep fluid element signatures^17^, once recalculated to the reservoir conditions. However, surface fluids have generally experienced boiling, which results in fractionation of the elements between fluid and vapor, and precipitation of scales that selectively sequester elements depending on the scale mineralogy^9^. Thus, the composition of reservoir fluids can differ drastically from the fluids sampled at the surface. In addition to fluids, actively exploited geothermal fields can also provide access to fresh and altered reservoir rock cores, cuttings and scale, and thus allow to fully explore the behaviour of elements in the deep system, as well as physical–chemical changes *en route* to the surface.

Down-hole devices have been developed and used to sample reservoir fluids directly and thereby enable the metal concentrations of pre-boiled geothermal fluids to be determined^9,18–20^. In the Taupo Volcanic Zone, New Zealand, geothermal reservoir fluids in an alkalic basalt hosted hydrothermal system have been shown to be metal-laden with up to 23 ppb Au, 2400 ppb Ag, and 4850 ppb As^9^. Basalt-hosted deep fluids in the Reykjanes peninsula in the plume-MOR setting of Iceland have lower metal concentrations despite their equivalent Cl content, with concentrations of up to 6 ppb Au, 34 ppb Ag, < 1 ppb As, and concentrations for Cu, Zn, and Pb at 16, 26, and < 1 ppb, respectively^10,21^. Nonetheless, the Reykjanes fluids result in enrichments in scale precipitates of up to 950 ppm Au and 2.5% Ag^22^.

In this contribution, we present compositions of surface and deep-sampled fluids from the Northern Volcanic Zone (NVZ) of Iceland: The newly developed Theistareykir geothermal field hosted in tholeiitic basalts erupted from a Holocene volcanic system^23^, and the adjacent Krafla field which is hosted in basalt and rhyolite^24^. Fluids have been characterized for a full suite of elements, O and H isotopes, and noble gases, allowing us to determine the mobility of elements in the deep reservoir, their fate during transport toward the surface, the sources of fluids and metals, and the impact of host rock geology. Direct compositional information on deep fluids is still extremely limited and this new data enables a deeper understanding of fluid-mediated element mobility in magmatic-hydrothermal systems with direct implications for global element cycling.

### Study area

The Northern Volcanic Zone (NVZ) is a unique geological observatory providing a window into a sub-aerial mid-ocean ridge–plume setting and its related magmatic-hydrothermal systems. In the NVZ, the interaction of the Icelandic mantle plume is small but present^5^. The NVZ has been the main zone of spreading in northern Iceland for the past 6–7 Myr^25^. The NVZ is composed of five NNE striking left-stepping *en échelon* volcanic systems^23^, amongst which the Theistareykir shield volcano and the Krafla caldera, both presently exploited for geothermal energy.

**Figure 1:**
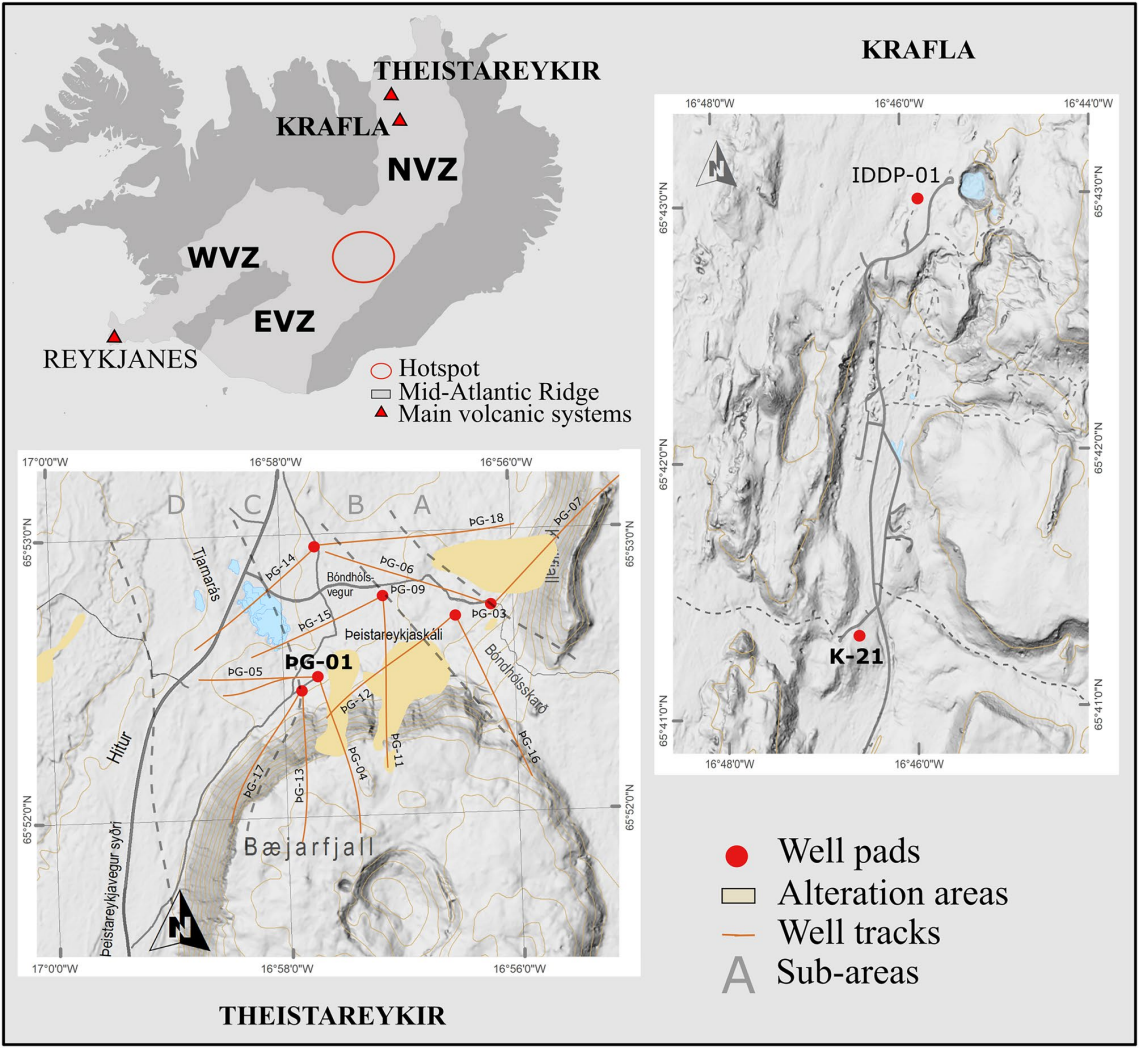
Simplified maps of the Theistareykir and Krafla geothermal fields with positions of the wells sampled for the deep fluids (ÞG-01 and K-21). *NVZ* Northern Volcanic Zone, *WVZ* Western Volcanic Zone, *EVZ*Eastern Volcanic Zone. The Digital Elevation Models (DEM) with wells position and tracks were provided by Landsvirkjun with their permission to use it for publication. The general map of Iceland was modified from Wikimedia. Creative Commons. by Chris.urs-o. https://commons.wikimedia.org/wiki/File:Volcanic_system_of_Iceland-Map-en.svg. The figure was assembled using Inskape 1.2. https://inkscape.org/release/inkscape-1.2/.

The Theistareykir geothermal field is newly developed with production started in 2017^26^. A total of 18 deep wells (ranging from 1500 to 3000 m depth) have been drilled to date and the geochemistry of extracted fluids is still under evaluation^5,27^. The Theistareykir reservoir is hosted by basalts (olivine-tholeiites with MgO contents between 7 and 16 wt% to picrites with up to 22 wt% MgO^28,29^. The helium isotopic composition suggests a dominant depleted mantle MORB-like source for volatiles, with a small amount (ca. 10%) of Icelandic mantle plume^5,30,31^. Natural surface geothermal manifestations include mud pots, fumaroles and steam vents. The recent start of exploitation implies that fluids have been minimally disturbed by exploitation and re-injection. Fluids sampled at the well-head are dilute, have chlorine as the dominant anion (from < 10 to *ca*. 600 ppm) and are near-neutral pH^27^. Downhole temperatures are among the highest recorded in potential production wells in Iceland at up to 380 °C in well ÞG-03^32^ (Fig. 1). The well ÞG-01 studied here is situated in the southern part of the geothermal field.

The adjacent Krafla is the most active part of the Northern Volcanic Zone, last erupting between 1975 and 1984^32–35^. The Krafla geothermal system is thought to be fed by a shallow magma chamber of binary rhyolitic—basaltic composition^36,37^. The Krafla field has been exploited since 1978 with reinjection of water starting in 2002^38^. As in Theistareykir, surface fluids are near-neutral pH^3^ and the highest fluid temperature measured in the area is 440 °C (well IDDP-1^39^; Fig. 1). The well K-21 studied here is located at the southern end of the field and is hosted by basalt.

Most Icelandic high-temperature geothermal fields are composed of near-neutral pH and Ca-HCO_3_ fluid. The Theistareykir and Krafla geothermal fields, however, exhibit some unusual fluid characteristics with fluid of alkali (Na–K)-chloride type. This type of fluid composition is unusual in Icelandic geothermal systems and reflects fluid that is more mature^40^ (e.g., older).

## Methods

Fluid samples were collected in August 2019 from wells ÞG-01 (Theistareykir) and K-21 (Krafla) at the well-head and at depths of 850 m for K-21, and for ÞG-01 at 1420 and 1600 m from the surface. Surface fluid samples were collected using a fluid separator while the well was producing, whereas the deep samples were collected when the well was shut-in to insert the deep sampler. Deep fluid samples were collected using the in-situ Ti-sampler of Brown and Simmons (2003)^9,17^. Temperature and pressure profiles were collected in the well immediately before sampling and indicate *ca.* 265 °C and 55 bar at 1420 m and 290 °C and 75 bar at 1600 m in well ÞG-01 and 235 °C and 50 bar at 850 m in K-21 (Supp. Fig. S1). Between 647 and 860 g of deep fluid was recovered, which was split into several aliquots; unfiltered, 0.45 µm filtered, and acidified with 5 µL of nanopure concentrated HNO_3_. Vials were fully filled so as to avoid any air entrapment. The pH was measured in the field with a freshly-calibrated glass electrode on the ÞG-01 deep fluid samples, and was around 2.5 compared to a near neutral pH for the wellhead condensed fluid. Following recovery of the fluid, the sampler was rinsed twice with freshly-prepared *aqua regia* (nanopure grade) to recover any precipitate, and this fluid was also collected. All fluids were stored in pre-cleaned PFA containers. Samples from the deep fluids were analysed for the first time for O and H isotopes according to the methodology described in ref.^5^. For anions by Ion Chromatography at ETS (Montreal, Quebec), and for major and trace elements by HR-ICP-MS at ActLabs (Ancaster, Ontario). Four units of fresh basalt were sampled across the Theistareykir area. Each unit was sampled at 3 different locations for a total of 12 samples. For Krafla, only the most recent lava flow (from the 1984 Krafla Fires) from the Leirhnjúkur area was collected. Samples of altered rock cuttings recovered from well ÞG-01 at 1600 m were provided by ÍSOR. These combined samples are taken here to represent the fresh basalt host rock of the geothermal reservoir and its altered equivalent. However, we acknowledge that basalt taken at the surface is likely to have experienced additional degassing compared to the intrusive rock below the surface. The fresh and altered samples were crushed and milled to below ~ 1 µm. This powder was then pressed into a pellet without additives and analysed by LA-ICP-MS at McGill University following the approach of ref.^41^. Full method details are given in the supplementary file.

## Results

**Figure 2:**
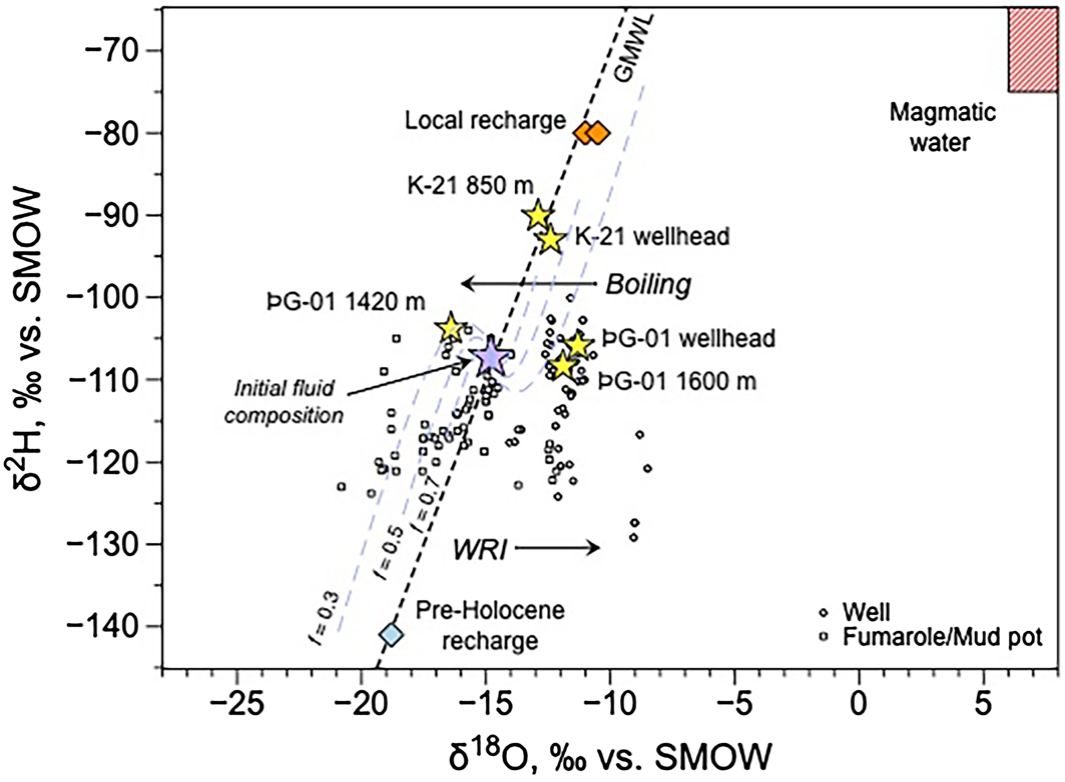
Plot of δ^2^H versus δ^18^O corrected for total discharge (TD) of the Theistareykir production fluids, mud pots, and fumaroles sampled over a 20-years period (data from this study and archived ÍSOR data, in addition to the values of the deep samples at Theistareykir and Krafla^5^; Table 1). The global meteoric water line (GMWL; dashed line) was calculated following ref.^44^. Pre-Holocene recharge composition is from ref.^42^, and the regional highlands recharge is from ref.^4^. Primary magmatic water composition is from ref.^45^. The dashed curves represent the evolution of δ^2^H_TD_ versus δ^18^O_TD_ in the vapor and residual liquid after phase segregation, starting from an initial composition (purple star). Water isotopic compositions evolve along these lines according to their final equilibrium temperature. The *f* values correspond to the fraction of residual liquid.

The stable isotope composition of surface fluids (δ^2^H, δ^18^O) from ÞG-01 and K-21, re-analyzed during the same campaign as the deep fluids sampling are consistent with literature data for these two fields^4,5,42^, reflecting contributions from a magmatic source, meteoric water, and at least one source of glacial water^4,42^, as also confirmed by noble gas isotopic signatures^5^. Deep fluids sampled at 1420 m (ÞG-01) and at 850 m (K-21) show δ^2^H values (− 108.4 ‰ and − 90.1 ‰ respectively) that are similar to their respective counterparts sampled at the well-head (Fig. 2). However, the shift towards the left side of the Global Meteoric Water Line (GMWL) shows that these deep fluids have undergone some boiling^43^ and are likely located close to the boiling horizon. In contrast, the 1600 m ÞG-01 sample, which is situated on the right side of the GMWL, shows no isotopic evidence for phase separation and this sample reflects the reservoir fluid below the boiling horizon, in agreement with the P–T curve for this well (Fig. S1a).

**Table 1:** Chemical composition of the deep and surface fluids (not corrected for vapor loss), fresh and altered rocks at Theistareykir and Krafla geothermal fields.

Elements (ppm)	Fumarole Theistareykir	ÞG-01 - 0 m	ÞG-01 - 1420 m	ÞG-01 - 1600 m	Theistareykir Fresh Basalt	Theistareykir most altered rock sample at 1600 m - ÞG-01	K-21 - 0 m	K-21 - 850 m	Krafla Fresh Basalt
P (bar)		8.87					11.04		
Enthalpy (kJ/kg)		1753					1129		
δ2H	− 80	− 105.90	− 103.83	− 108.44	*n.a*	*n.a*	− 93.00	− 90.11	− 117.00
δ18O	− 6.5	− 11.31	− 16.43	− 11.92	4.7	*n.a*	− 12.40	− 12.94	− 5.00
Cl	3.01	96.26	40.00	68.00	*n.a*	*n.a*	210.76	130.00	*n.a*
SO4	513.97	12.17	524.00	250.00	*n.a*	*n.a*	52.10	1137.00	*n.a*
Li	0.002	0.12	0.07	0.09	2.96	5.90	0.24	0.16	6.82
Na	5.01	107.10	64.97	98.35	11,451.21	18,845.90	198.69	137.22	9901.23
K	0.63	21.84	12.53	18.00	567.53	2522.20	30.26	18.92	2416.42
Rb	0.002	0.11	0.06	0.10	1.20	5.35	0.0003	0.10	3.59
Cs	*n.d*	0.004	0.003	0.004	0.02	0.00	*n.d*	0.01	0.34
Be	*n.d*	*n.d*	0.0001	0.0001	*n.d*	*n.d*	*n.d*	0.0001	0.66
Mg	10.65	0.03	0.54	0.40	72,242.74	40,515.70	0.001	1.11	35,589.00
Ca	14.78	0.23	1.50	0.87	89,875.31	*n.d*	1.13	3.20	59,730.44
Sr	0.03	0.01	0.01	0.004	104.86	249.85	0.0	0.03	167.15
Ba	0.018	0.00012	0.05	0.02	21.99	53.35	0.0004	0.08	71.54
Ti	0.02	0.001	0.11	0.52	4335.50	7703.45	0.001	0.36	11,499.23
V	0.055	0.003	0.02	0.02	252.13	255.20	0.002	0.04	249.87
Cr	0.032	0.00002	0.03	0.05	828.72	233.25	0.00002	0.15	105.73
Mn	0.13	0.002	0.43	0.08	1378.93	1541.20	0.0002	1.19	1780.72
Fe	14.91	0.003	6.68	14.60	76,765.05	71,459.45	0.01	44.63	97,696.71
Co	0.007	0.00003	0.01	0.04	63.00	57.55	0.0	0.02	34.35
Ni	0.032	0.0002	0.03	0.43	285.72	63.90	0.0001	0.67	75.59
Cu	0.0015	0.002	0.11	0.62	121.90	27.50	0.0	1.35	119.86
Zn	0.062	0.002	0.44	0.25	77.84	92.60	0.001	3.34	113.25
Zr	0.0005	0.0001	0.001	0.001	33.87	127.40	*n.d*	0.001	130.79
Nb	*n.d*	*n.d*	0.0003	0.0001	2.07	7.90	*n.d*	0.0002	10.33
Mo	*n.d*	0.001	0.03	0.05	0.28	3.50	0.001	0.05	1.05
Ag	*n.d*	*n.d*	0.01	0.01	0.02	0.00	*n.d*	0.01	*n.d*
Cd	*n.d*	0.000024	0.001	0.004	0.10	0.10	0.0	0.02	*n.d*
Hf	*n.d*	*n.d*	0.00004	0.00003	0.95	3.80	*n.d*	0.00003	3.52
Ta	*n.d*	0.000001	0.0001	0.00002	0.43	0.45	*n.d*	0.00002	0.26
W	*n.d*	0.01	0.002	0.003	6.57	0.90	0.00003	0.001	0.78
Re	*n.d*	0.0002	0.000001	0.0001	*n.d*	*n.d*	*n.d*	*n.d*	0.0002
Hg	*n.d*	0.00015	0.001	0.004	0.03	*n.d*	0.0	0.002	*n.d*
Al	21.49	1.78	1.11	1.91	71,970.92	91,472.60	1.34	3.18	74,363.00
Ga	0.001	0.005	0.002	0.005	6.70	20.35	*n.d*	0.003	22.61
Ge	0.002	0.03	0.01	0.02	*n.d*	*n.d*	*n.d*	0.02	2.09
In	*n.d*	*n.d*	0.00001	0.000003	0.04	*0.10*	*n.d*	0.0	0.03
Sn	*n.d*	*n.d*	0.002	0.001	0.43	0.80	*n.d*	0.01	0.68
Sb	*n.d*	0.000005	0.10	0.05	0.02	0.10	*n.d*	0.04	0.06
Tl	*n.d*	0.000038	0.0001	0.0002	0.005	0.00	*n.d*	0.0001	0.03
Pb	*n.d*	0.00002	0.01	0.01	5.52	*1.25*	0.00001	0.05	1.29
Bi	*n.d*	0.000024	*n.d*	*n.d*	0.05	0.00	*n.d*	*n.d*	0.02
B	0.004	1.15	0.58	0.88	*n.d*	*n.d*	0.81	0.63	*n.d*
Si	73.02	351.00	175.90	252.96	216,723.32	227,061.60	272.00	178.70	227,406.58
As	*n.d*	0.003	0.03	0.03	0.07	0.35	0.01	0.03	0.08
Se	*n.d*	0.001	0.03	0.24	0.09	0.150	*n.d*	0.01	*n.d*
Te	*n.d*	0.001	0.003	0.01	*n.d*	*n.d*	*n.d*	0.005	1.88
Sc	0.022	0.001	*n.d*	*n.d*	35.63	33.70	*n.d*	0.0004	25.94
Y	0.008	*n.d*	0.0002	0.0003	15.44	29.60	*n.d*	0.001	47.06
La	*n.d*	0.00001	0.0002	0.0002	2.01	*7.95*	*n.d*	0.0003	7.69
Ce	*n.d*	0.00002	0.0003	0.0004	5.59	14.85	*n.d*	0.0007	15.24
Pr	*n.d*	*n.d*	0.00003	0.00005	0.87	2.40	*n.d*	0.0001	10.64
Nd	*n.d*	*n.d*	0.0002	0.0002	4.53	11.35	*n.d*	0.0004	12.61
Sm	*n.d*	*n.d*	0.00003	0.0001	1.58	*4.10*	*n.d*	0.0001	4.28
Eu	*n.d*	*n.d*	0.00001	0.000017	0.65	1.40	*n.d*	0.00003	2.11
Gd	*n.d*	*n.d*	0.00004	0.0001	1.98	4.70	*n.d*	0.0001	5.15
Tb	*n.d*	*n.d*	0.000003	0.00001	0.39	1.10	*n.d*	0.00002	0.76
Dy	*n.d*	*n.d*	0.00003	0.00005	2.63	4.90	*n.d*	0.0001	4.32
Ho	*n.d*	*n.d*	0.000003	0.00001	0.58	1.15	*n.d*	0.00002	1.22
Er	*n.d*	*n.d*	0.00002	0.00003	1.65	3.30	*n.d*	0.0001	3.91
Tm	*n.d*	*n.d*	0.000001	0.000002	0.25	0.55	*n.d*	0.00001	0.53
Yb	*n.d*	*n.d*	0.00001	0.00002	1.68	3.05	*n.d*	0.00005	4.31
Lu	*n.d*	*n.d*	0.000001	0.000002	0.25	0.60	*n.d*	0.00001	0.40
Th	*n.d*	*n.d*	0.0002	0.0001	0.14	0.60	*n.d*	0.0001	1.15
U	*n.d*	*n.d*	0.00002	0.00004	0.04	0.20	*n.d*	0.0001	0.27

*n.a.* : not analyzed.

*n.d.* : not detected.

At both sites, the well-head fluids are dilute (TDS ~ 350 mg/L), near-neutral solutions dominated by Na–K–Cl. The deep fluids have a higher TDS (~ 1650, ~ 830 and ~ 700 mg/L for 850 m at K-21, and 1420 and 1600 m at ÞG-01, respectively). The Theistareykir deep fluids have an acidic pH between 2 and 2.6. The pH of the K-21 deep fluid was not directly measured, but the charge balance shows a cation deficit that also points towards an acidic fluid. This would be consistent with the acidic character of these waters in some portions of the field^40,46,47^.

Calcium, Mg, Fe, Sb, Te, Zn, Cu concentrations in the deep fluids at Theistareykir are the same order of magnitude as deep fluids from other geothermal fields including Iceland (Reykjanes), New-Zealand (Wairakei), Japan (Kakkonda), USA (Geyser), and Mexico (Los Humeros)^1^. Aluminium is distinctly higher, whereas As is lower than these examples. For Na, K, and Cl, the Theistareykir deep fluid concentrations are between those found in conventional and supercritical reservoir fluids.

**Figure 3:**
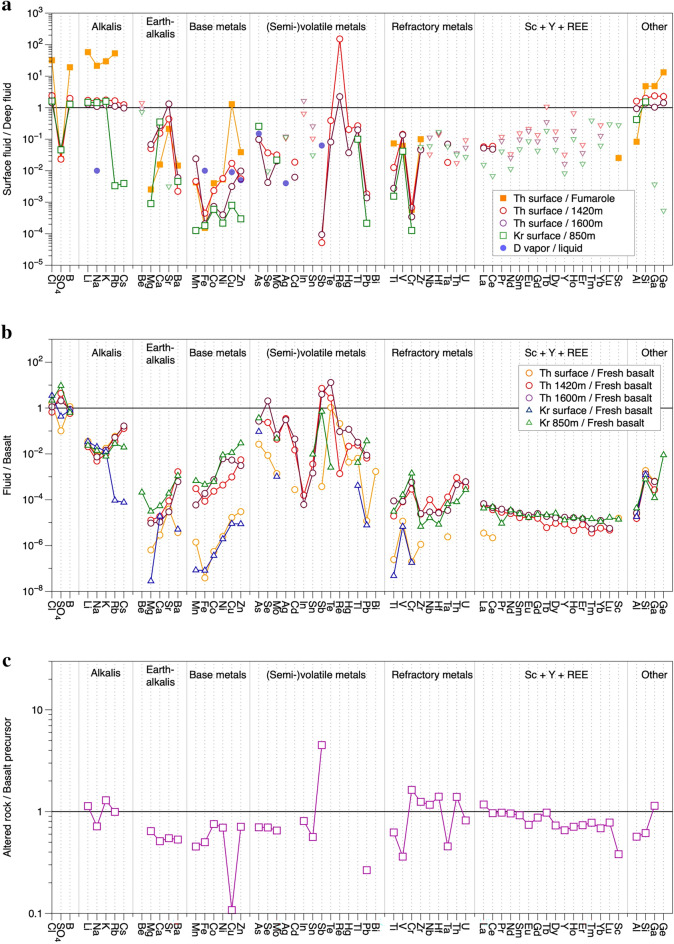
(**a**) Diagram of the chemical composition of the fluids sampled at the surface (as condensed vapor) normalized to the deep fluids at depths of 1420 and 1600 m for Theistareykir and 850 m for Krafla. The composition of Theistareykir acidic fumarole fluids is also shown, as are vapor/fluid partition coefficients from ref.^49^. The maximum value is shown as a triangle for elements where the concentrations in the surface fluid were below the detection limit, calculated from the respective detection limit. (**b**) Composition of the fluids normalized to their respective fresh local basalt. (**c**) Composition of the altered rock cuttings at a depth of 1600 m, normalized to the fresh local basalt for Theistareykir. The elements have been grouped first by their geochemical behaviour and then their mass.

Figure 3a shows the composition of the surface fluids normalized to their deep counterparts. The behaviour of the elements in the fluids from Theistareykir and Krafla wells is largely consistent, with an overall larger difference between surface and deep fluid element concentrations for Krafla, as a combined result of lower surface and higher deep fluid concentrations. The behaviour of Rb, Cs, Mg, Sr, Ga and Ge differs between Krafla and Theistareykir and mainly reflects lower concentrations for these elements in Krafla surface fluids. This suggests the presence of one or more additional phases in the Krafla scales that sequester these elements. Element concentrations are mostly 1–3 orders of magnitude lower in surface fluids than fluids at depth (Fig. 3a) except for Cl, B, the alkali elements, Sr, Al, Si, Ga and Ge that are essentially the same or somewhat enriched, Re that is strongly enriched in the surface fluid, and the semi-volatile metals As, Te, Hg and Tl that are less depleted than most. Sulphate, Mg, Ba, the base metals, Sb, Pb, Ti and Cr are particularly depleted. The observed behaviour of elements is similar to that observed for surface versus deep fluids (directly sampled and fluid inclusions) from the Reykjanes peninsula in Iceland^48^.

Figure 3b shows the fluid compositions normalised to the fresh Theistareykir and Krafla basalts. The semi-volatile metals are either completely enriched or relatively enriched in the fluid compared to other metals and to the host basalt. The alkali elements, Rb and Cs in particular, are less depleted than the alkali-earth elements. Copper, Zn and Ni are enriched in the fluid relative to Co, Mn and Fe. On the other hand, the refractory elements (e.g. Nb, Zr, Ti, Hf and Al) and the REE are strongly depleted in the fluids compared to the fresh rock, with a small but consistent LREE to HREE decrease.

The altered rock cuttings from the Theistareykir well at 1600 m show an overall enrichment in all the elements except Cu and Pb compared to the fresh Theistareykir basalt, especially for the most refractory elements including Zr, Nb, Hf, Th, U and the REE. These refractory elements have a common enrichment factor of *ca.* 2. We interpret this to reflect residual enrichment of these elements during alteration as a result of their immobile nature, and we therefore normalise the altered rock composition to the content of these elements in the fresh basalt^50^. The normalized altered over fresh composition is shown in Fig. 3c, and indicates approximate conservative behaviour for the alkalis, refractory metals and LREE, and depletion in the earth alkalis, base and (semi)-volatile metals, and the HREE. Copper is strongly depleted, whereas Sb is highly enriched. These trends broadly follow alteration-associated element re-distribution in Reykjanes reservoir rocks^51^, despite the Reykjanes fluids having much higher Cl-contents.

### Evolution of fluids in the geothermal well

**Figure 4:**
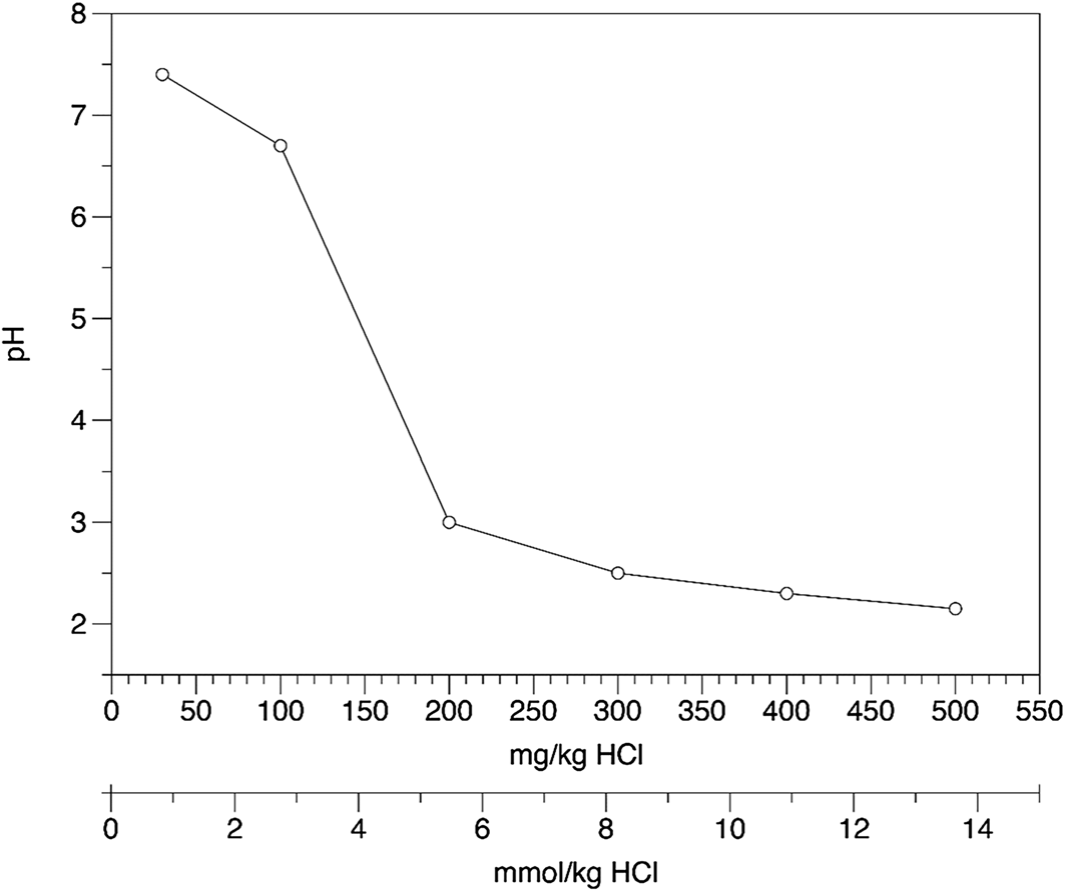
Evolution of pH depending on the HCl concentration of the fluids.

The temperature and pressure profile in the wells (Fig. S1) indicate that the deepest parts are below the boiling horizon, and the resident fluid at depth is a 1-phase solution. As this fluid rises up the well, it reaches the boiling horizon and splits into a vapor and residual liquid phase. Whereas the vapor rises to the surface, the denser residual liquid is thought to descend in the well, mix with the deeper fluid, and/or flow laterally out of the well. Surface fluids are therefore predominantly composed of the vapor fraction. During ascent to the surface additional fluid or steam can enter the well and mix with the vapor, especially when the well is shut-in to permit the deep fluid sampling. An increase in T indicates an influx of hot fluid or steam, whereas a drop in T shows an inflow of colder water (e.g. groundwater). For well ÞG-01, an inflow of liquid water is noticeable around 1275 m depth in the P–T profile, which is well above the boiling horizon (Fig. 4).

The fluids collected at the surface exhibit a near-neutral pH, and given that boiling generally produces a vapor with a lower pH than the 1-phase source fluid^52^, this indicates that the deep fluids must also have a near-neutral to slightly alkaline pH. This is consistent with the secondary mineral assemblage (epidote + chlorite + albite + prehnite ± K-feldspar) observed for Krafla and in the nearby Namafjall geothermal field^53^. However, the fluids sampled at depth—when the wells were shut-in—were acidic (pH 2–2.6). We interpret this discrepancy to reflect influx of a low pH fluid into the well, likely a condensate of acidic gases (e.g., SO_2_, H_2_S, CO_2_, and HCl) into groundwater. Such hypogene acid fluids have also been reported in the Tiwi field in the Philippines^54^ and the Miravalles field in Costa Rica^55^. These acidic gases may have three origins: (1) they could be derived directly from the magmatic intrusions that are associated with the Krafla and Theistareykir geothermal fields but this would result in significant δ^2^H and δ^18^O shifts of the hydrothermal fluids that are not observed, (2) they could be generated by the condensation of vapor on the way to the surface, but this process would unlikely be able to lower the pH to 2.5 (rather closer to 5–6), or (3) represent shallow condensation of the acid vapor formed in secondary boiling and which is emitted in fumaroles in the Theistareykir field close to well ÞG-01. Given that these geothermal waters have a low capacity of buffering the pH due to their low element concentrations and content of dissolved CO_2_, even a small amount of acidic water mixing with deep fluids can result in a significant drop of the pH (Fig. 4).

In this interpretation, the deep fluids do not represent a pristine deep fluid composition. It is unclear to what extent the fluid has been modified by addition of elements from the acidic fluid influx, or by interaction between the acidified fluid and its now-in-disequilibrium host rock mineralogy. The wells were only shut-in for few hours prior to the deep sampling so fluid-rock interaction may not yet have significantly affected the element concentrations. Indeed, carbonates, present in cuttings as a secondary phase, would be expected to dissolve in the acidified fluid, but Ca and Sr concentrations follow the overall trends of the earth-alkali elements in Fig. 3b, and mimic the trend for the surface fluids. The acidic surface fumarole fluids sampled in Theistareykir are depleted in most elements compared to surface and deep well fluids with the exception of S, Mg, Ca, Sr, Mn, Fe and Al (Fig. 3b). Addition of elements from a fumarole-like fluid would thus result in higher concentrations in the deep fluid of these elements, yet this signature is not observed. We therefore conclude that the impact of host-rock re-equilibration and element addition was minimal.

### Metal behaviour patterns

The fluid compositions reflect multiple processes at work in the geothermal system, including a primary magmatic input, dilution by modern and ancient groundwater, water–rock interaction with associated element mobilization and sequestration, boiling and its associated element partitioning, and scale formation and its associated element sequestration. The elements and the media analyzed here respond variably to these processes, thereby potentially allowing for their impact to be identified and quantified.

The basalt-normalized fluid compositions show higher values for the semi-volatile metals, and particularly low values for the refractory metals and the REE. This can be interpreted as preferential mobilization of the semi-volatile metals during water–rock interaction, addition of the semi-volatile metals from a different source, and/or selective precipitation of the refractory metals and REE. The altered-over-fresh rock composition shows more or less conservative behaviour for the REE (Fig. 3c), and water–rock interaction therefore does not significantly impact these elements in the fluid. The same can be argued for the majority of the refractory metals and alkalis, but the earth alkalis, base metals and semi-volatile metals are depleted in the altered rock and are therefore released during water–rock interaction. In contrast, Sb is highly enriched in the altered rock and is preferentially sequestered. This is mirrored in the singular depletion in Sb for surface fluid relative to the deep fluid (Fig. 3a). The relative depletion for the 3 groups of elements is approximately the same. However, their behaviour in the fluid/basalt plot is distinctly different, with the earth alkalis the lowest, and the (semi)-volatile metals the highest (Fig. 3b). This requires a source other than water–rock interaction for the base and (semi)-volatile metals. The semi-volatile metals are preferentially enriched in magmatic gases, as are the base metals albeit to lower extent^52–58^. We therefore interpret this additional source to be magma degassing and for the behaviour of these elements in the Theistareykir and Krafla deep fluids to reflect a strong contribution from the magmatic system. This is supported by noble gases, which indicate a direct transfer of material from the magma to the geothermal fluid, as evident in the ^3^He/^4^He ratio^5^.

The fluids are enriched in LREE over HREE. This is not the result of water–rock interaction, because the LREE act conservatively in the altered rocks whereas the HREE, Y and Sc are leached (Fig. 3c). Rather, we interpret this to reflect the higher stability constants for LREE over HREE-species in the fluid^20^, and therefore a stronger mobilization of the LREE from the magma.

The differences between surface and deep fluids can be expected to mainly reflect boiling and the associated element partitioning between vapor and residual liquid, with the surface fluid dominantly, or even exclusively, reflecting vapor. The volatile metals are less depleted than, for example, the base metals in the surface fluids, which is consistent with their preferential fractionation into the vapor, and the element pattern in surface *vs.* deep fluid follows, to some extent, vapor–liquid partition coefficients^49^ (Fig. 3a). Concentrations of Cl, B, the alkali metals, Al, Si, Ga and Ge are enriched in the surface fluids. Several processes can contribute to this enrichment, including (1) the loss of steam from depth to the surface, thus residually concentrating the surface fluids compared to the deep fluids, and (2) metastable persistence of elements above their solubility, aided by the thermal state of the system—excess enthalpy—and the type of fluid—NaCl-type rather than steam-heated acid-sulphate waters^59^.

The base metals are among the most depleted in the surface fluids compared to the deep fluids (Fig. 3a), yet they are released during water–rock interaction. We therefore interpret this to reflect precipitation in scale along the walls of the well or in the host rocks. Well scales of geothermal sites throughout Iceland contain silica, iron-silicate, iron-magnesium-silicate as well as pyrite, pyrrhotite, magnetite and other metal sulphides^39,40,59,60^. The sulphides, in particular, can be an important host for base metals, as observed in sulphides from Reykjanes well fluid precipitates^19^.

### Implications for element mobility in magmatic-hydrothermal systems

Geothermal production fields in magmatic settings provide a unique opportunity to study the behaviour of elements in magmatic-hydrothermal fluids, in particular by providing direct access to deep fluids. The Theistareykir and Krafla fluids provide a consistent story of metal enrichment and depletion patterns. The two fields share common background conditions for the mobilisation of metals in deep geothermal fluids with similar reservoir host rocks and secondary mineralogy, fluid temperatures, and the same local meteoric water and regional groundwater. These conditions are typical for basaltic magmatic settings, and we therefore posit that the results obtained here are representative for such systems, showing that element concentrations reflect a magmatic degassing input variably modified by water–rock interaction. There are significant changes between the deep fluid composition and that at the surface, in absolute concentrations, but especially in the element signature as a result of boiling and scale precipitation. This means that element content and pattern from surface fluids cannot be interpreted as directly reflecting the deep reservoir fluid composition, and the recalculation commonly applied cannot correct for this.

## Supplementary Information

Supplementary Information.

## Data Availability

The datasets generated during and/or analysed during the current study are not publicly available due to ongoing research but are available from the corresponding author on reasonable request.
