# Supplementary material for: The behaviour of metals in deep fluids of NE Iceland
Source: Sci Rep. 2022 Dec 19;12:21952. doi: 10.1038/s41598-022-26028-x (PMC9763496; doi:10.1038/s41598-022-26028-x)
Supplement: Supplementary file 1 — Supplementary Information. [file 41598_2022_26028_MOESM1_ESM.pdf]

The Behaviour of Metals in Deep Fluids of NE Iceland

M. Saby, V. van Hinsberg, D. L. Pinti, K. Berlo, B. Gautason, Á. Sigurdardóttir, K. Brown, O. Rocher

Supplementary Information

The Supplementary Information includes:

- Deep Sampling
- Sample Preparation and Analysis
- Figures S-1
- Table S-1
- Supplementary Information References

Sampling of Deep and Surface Fluids, and Fresh and Altered Rocks

Two vertical wells were sampled for this study: one in the Theistareykir Geothermal Field, with a bottom depth of 1953 m (ÞG-01), and the second in the Krafla Geothermal Field, with a bottom depth of 1200 m (K-21). Both wells are active production wells and were shut-in several hours prior to sampling to allow for the deep sampler to be inserted into the well. The wells were logged with a Kuster K-10 tool immediately prior to sampling to determine P and T profiles (Fig. S1a,b). Deep fluid samples were collected using a pacified Ti-metal sampler developed by Kevin Brown and described in detail in Brown and Simmons, 2003 and Simmons et al., 2016[1,2]. The evacuated sampler was lowered into the well with a steel cable the length of which was monitored to determine the depth of the sampler. The sample was collected by physically puncturing a rupture disc at the desired depth with the pressure-differential forcing the fluid into the sampler. This same fluid pressure subsequently pushed a spring-loaded valve closed during ascent to keep the sample chamber sealed. At the surface, the sampler was quenched in water and then opened to recover the fluid.

A total of three samples were collected: two from ÞG-01 (1420 m and 1600 m below the surface) and one from K-21 (850 m). The 1420 m sample from ÞG-01and the 850 m sample from K-21 have been collected close to the boiling horizon while the 1600 m from ÞG-01was collected below the boiling horizon as indicated by the down-hole pressure and temperature measurements (e.g., Fig. S-1a,b). 860.2 g of liquid was recovered for K-21, 647.37 g for well ÞG-01 at 1420 m, and at 1600 m, 841.84 g of fluid was recovered. After collecting the fluid, the sample was divided into three sub-samples: (1) an unacidified aliquot for the analysis of anions and water isotopes, (2) a sample acidified with ultra-pure HNO_3, and (3) a sample filtered through a 0.45 µm disposable filter and acidified with ultra-pure HNO_3..Sample vials were fully filled to avoid entrapping any air. Then, the titanium sampler was rinsed twice with a freshly-prepared, concentrated solution of trace-metal grade aqua regia (~ 40 ml) to dissolve any precipitates followed by ~ 40 ml of distilled water, and these rinse solutions were collected. All solutions were stored separately in PFA or PFA-lined HDPE bottles. The original element content was then reconstituted from all the subsamples after being analysed separately (see below).

A method blank has been determined in the study of geothermal wells in New Zealand in which this exact sampler was filled with deionized and lowered close to 1 km depth (255 °C) for 10 min. The blank solution was recovered and the sampler washed with the aqua regia and deionized water, with these rinse solutions added to the blank. These method blanks consistently returned concentrations at least 10 times lower than the lowest measured concentration in our samples[1,2].

Surface fluids were sampled at the well head of ThG-01 and K-21 while the well was producing. Fluids were collected in HDPE 30 mL bottles, unfiltered for the analysis of anions and 0.45 µm filtered and acidified with 5 µL of nanopure concentrated HNO3 for the analysis of cations. Bottles were fully filled to avoid air entrapment.

Four units of outcropping basalt were sampled across the Theistareykir area to represent the fresh basalts in the subsurface of well ÞG-01. Each unit was sampled at 3 different locations for a total of 12 samples. Only one lava sample has been taken for Krafla, on the most recent lava flow, from the Leirhnjúkur area. ÍSOR provided a sample of rock cuttings from 1600 m of Theistareykir well ÞG-01 consisting of fragments of variably altered rock. The cuttings were washed in deionized water and air dried, and then split into 8 sub-samples to create a mixing array with variable alteration. The fresh Theistareykir lava at the end of this mixing array is used here as the precursor basalt and as the normalizing basalt for Theistareykir in Figure 3.

Sample Preparation and Analysis

Surface Fluids

Chemical analyses of anions and cations were carried out at the Krafla Power Station's laboratory using ICP-SFMS and ICP-AES, and were conducted by Helgi A. Alfreðsson and Júlía Björke of Geochemý. The heavy metals in the fluids were analyzed by ALS Global in Sweden. Full details can be found in Hauksson, 2020[3].

The volatile metals (Hg, Bi, Cd, Sb and Tl) were analyzed at McGill University by ICP-MS using an optimized method with long dwell times to obtain low detection limits. The McGill ICP-MS analyses were conducted using a Thermo-Finnigan iCAP-Qc ICP-QMS. All samples and standards were analysed in triplicate. The precision for these analyses is better than 7.7 % as determined from the triplicate analyses.

Deep Fluids

The deep fluid composition was reconstituted from the direct fluid samples and the compositions of the sampler rinse solutions. Moreover, precipitates were observed in the direct deep fluid sample vials after a few days, likely resulting from cooling, degassing and associated REDOX changes. The elements in these precipitates were also determined and added to the dissolved element content of the fluids. The following analytical protocol was applied:

STEP 1

10 mL of the supernatant fluid of the deep fluid samples was extracted after allowing the precipitates to settle for several weeks and ensuring that all precipitates were at the bottom of the vials and nothing was in suspension.

The remaining supernatant fluid was transferred to another Teflon vial for storage. As much fluid as possible was extracted, but none of the precipitate. The vials were subsequently dried in a desiccator overnight and weighed before and after to determine the amount of remaining fluid. It is assumed that only water is lost in this drying.

12 mL of an aqua regia – HF mixture was added to the precipitate to bring it into solution, and the closed vial heated to 80˚C for two and a half days and weighed before and after to make sure no fluid was lost. This acid was prepared from nano-pure HF, aqua regia and water, in the proportion 1:1:1. No visible solids remained at the end. The resulting solution was transferred to a Teflon vial and the original vial cleaned and weighed to determine the total amount of sample it had originally contained.

STEP 2

A small amount of nano-pure HF was added to the aqua regia rinse solution to obtain the same HF concentration as in the precipitate-dissolution solution above. The fluid was heated at 40˚C overnight on a hot plate to dissolve any silicate precipitates, and weighed before and after to make sure no fluid was lost. No visible solids remained after the heating.

STEP 3

The various solutions were analysed by ActLabs as follows;

The undiluted supernatant fluid sample was analysed by method HydroChem using ICP-MS, ICP-OES and HR-ICP-MS.

The aqua regia precipitate digest solution and aquea regia sampler rins were analysed by method ultratrace 2 using ICP-OES and ICP-MS analysis, including a blank of the digest solution.

STEP 4

The masses of the various fluid components were used to reconstitute the fluid from the partial analyses and determine its original total composition. The blank digestion solution only had measurable concentrations of Si, Al, Ti, V, Co, Zr, Nb, Sb, Cs, Ce, Ta and Th, and in all cases concentrations of these elements in the blank were low compared to the concentrations in the digested precipitates. Blank subtraction was applied to remove this blank contribution. The precipitates contain base metals (Fe, Co, Ni, V, Cr) and refractory elements (Ti, Al, REE), but no significant concentrations of soluble elements including the alkalis, as would be expected. For most elements, the final concentration predominantly reflects the supernatant fluid.

Precision and accuracy were determined by ActLabs on in-house and international reference samples analysed with our samples. These indicate precisions below 7.5% for all the elements except for B (39.2%). The accuracy is better than 12% for all the elements except for Li (388%), Zn (19.5%) and Se (34.2%).

Pellet analyses of altered and fresh rocks

A set of 20 samples (12 for the fresh rocks and 8 for the altered rocks) was analysed using a pellet laser-ablation ICP-MS method after Garbe-Schönberg and Müller (2014)[4]. Fresh rock samples were hand crushed wrapped in brown Kraft paper with a steel hammer and an aliquot was dry milled in a WC ring mill. The milled powders and selected reference materials were then wet milled in a Micro Mill Pulverisette 7 WC ball mill to a nanoparticulate powder. Mixtures of 2 g powder and 5 mL nanowater were milled in a WC vessel for intervals of 3 minutes, with 1-minute pauses over a period of 40 minutes, at 500 Hz. Between samples, the milling vessels were cleaned using high purity ethanol and fresh quartz powder was milled between each sample. Altered samples were hand crushed in an agate mortar to below ~1µm. All the powdered samples were then dried in an oven at 60°C and re-homogenised with high purity ethanol in an agate mortar. A 2 to 5 g aliquot of this fine-milled powder was pressed into a 10 mm diameter, 3 to 5 mm tall pellet in a polished steel piston-cylinder at a pressure of 20 MPa without an additive and was subsequently stored in a desiccator. The resulting pellets are hard, robust, and encased in aluminium cups, with a smooth upper surface.

Pellets were analysed by laser ablation ICP-QMS on a NewWave 213 nm Nd:YAG laser-ablation system coupled to a Thermo Finnigan iCapQc ICP-QMS. The ablated material was transferred to the ICP-MS in a 800 mL/min He flow, mixed with Argon prior to injection of material into the plasma. Concentrations for 60 elements were determined. The surface of each analysis spot was pre-ablated before analysis, to obtain a clean surface. Five spot replicates were analysed per sample at 160 µm spot size, 20 Hz laser repetition rate, 120 s dwell time, a 40 s washout, and a laser fluence of 4.2 J/cm^2. Analyses of NIST SRM 610 glass bracketed the analyses and were used to correct for drift, using Mg as the internal reference element. The data were processed using Iolite (v.2.5, Paton et al.. 2011) [5] with integration windows set using the Mg-divided signals. A background window was defined before and after each analysis with linear interpolation between these used to subtract the background for each integration window. Analyses in the same session of pelletized reference materials were used to construct a multi-standard calibration curve, following Peters and Pettke (2016)[6]. For the fresh basalt, three reference materials of varying bulk rock composition were included in this calibration, bracketing the compositions of the samples: PCC1, Jb_1a and Ja_1. For the altered basalt samples, 15 reference materials of varying bulk rock composition were included in this calibration, bracketing the compositions of the samples: PCC1, Jb_1a, Ja_1, BCR-2, BHVO-2, SY-3, SY-4, TILL-1, JR-1, JR-2, SARM2, SARM3, SARM44, NGRI-UMR, and OOKO201. Certified values for these reference materials were taken from the GEOREM database, using the GEOREM

preferred values where available[7]. For the fresh rocks, the precision on the analyses as determined from duplicates is better than 22% for elements less than 10 ppm, 8% for elements between 10-100 ppm, and 12% for elements that are 100 ppm+. For the altered rocks, the precision on the analyses as determined from duplicates is better than 10% for all elements except Mo (11%). For the fresh rocks, the accuracy, as determined by the residuals of the calibration on the reference material, is better than 21% on all elements. For the altered rocks, the accuracy is better than 15% on all elements, except B at 30% and Se at 36%.
Major element compositions were determined by powder EDS-XRF on an Olympus X-5000 instrument. Approximately 5 g of milled material was placed in a plastic cup with a 6 µm Mylar window and counted in air at 35 and 10 kV incoming X-Ray beam for 180 s each. Fourteen reference materials were included to construct a multi-standard calibration curve, and precision was determined from duplicate analyses on samples and reference materials. Certified values for the reference materials were taken from the GEOREM database (http://georem.mpch-mainz.gwdg.de/ accessed Oct 2020). The precision of the analyses is better than 2.5% relative.

Concentrations for Pb, Cl and S in the fresh Theistareykir and Krafla basalts are from the GEOROC database (http://georoc.mpch-mainz.gwdg.de/ - extracted Jan 2022). Rhenium is from Schilling and Kingsley (2017)[8] and Se + Te are from Forest et al. (2017)[9] both for sub-aerial Reykjanes basalts. For B, a value of 1 mg/kg was assumed based on the typical MORB range of 0.5 to 1.5 mg/kg.

Supplementary Figures

a

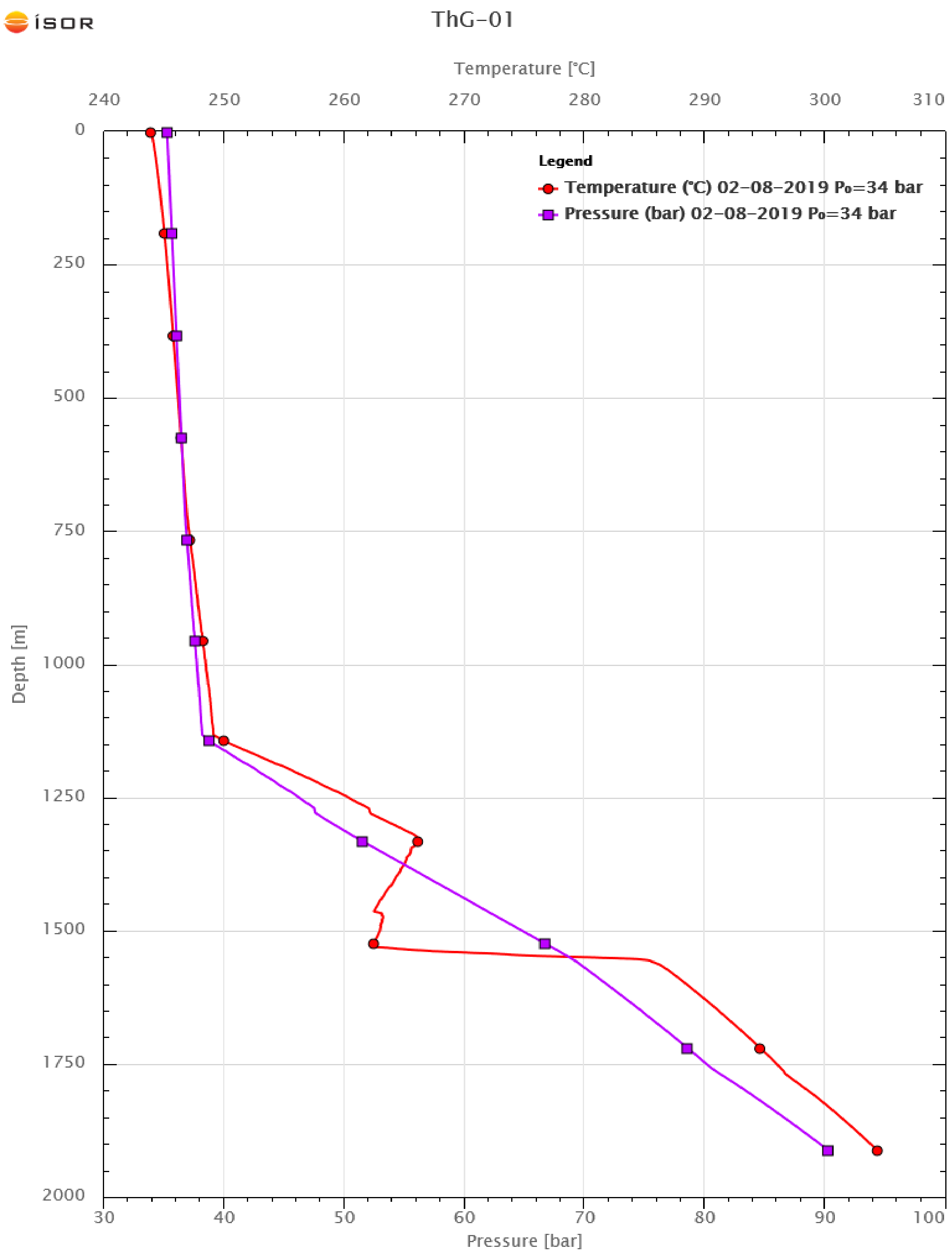

b

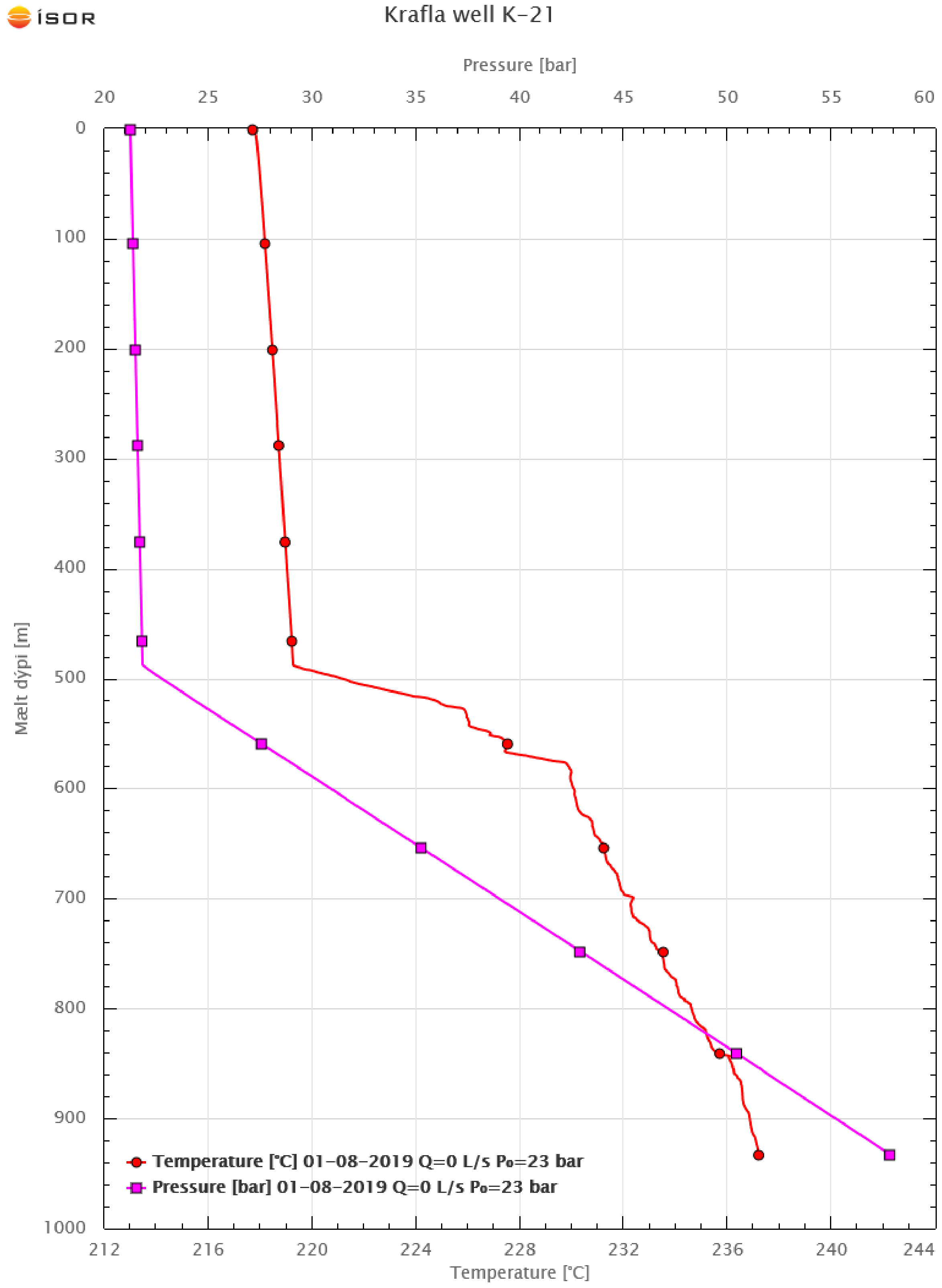

Figure S-1 Profile of pressure and temperature in wells ÞG-01 **(a)** and K-21**(b)** just before the sampling of deep fluids.

Table S-1. Uncertainties on the raw data

Elements (ppm)	ÞG-01 - 0 m	ÞG-01 - 1420 m	ÞG-01 - 1600 m	Theistareykir Fresh Basalt	Theistareykir Most Altered Rock sample at 1600 m - ÞG-01	K-21 - 0 m	K-21 - 850 m	Krafla Fresh Basalt
δ2H	0,14	0,50	0,20	*n.d.*	*n.d.*	0,20	0,30	n.d.?
δ18O	0,02	0,10	0,03	n.d.?	*n.d.*	0,10	0,03	n.d.?
Cl	2,00	2,00	2,00	*n.d.*	*n.d.*	2,00	2,00	*n.d.*
SO4	0,20	0,20	3,50	*n.d.*	*n.d.*	0,20	3,50	*n.d.*
Li	0,00	0,00	0,00	0,19	0,00	0,00	0,00	0,91
Na	10,00	5,88	8,90	833	497,10	15,00	12,41	1267,14
K	1,60	1,50	2,16	40,6	135,50	2,20	2,27	261,52
Rb	0,0027	0,0027	0,0041	0,089	0,05	0,0027	0,0043	0,45
Cs	*n.d.*	*n.d.*	*n.d.*	0,00037	*n.d.*	*n.d.*	*n.d.*	0,07
Be	*n.d.*	0,0000061	0,0000093	*n.d.*	*n.d.*	*n.d.*	0,000012	1,45
Mg	*n.d.*	0,039	0,029	4047	*1306,70*	*n.d.*	0,080	2960,07
Ca	*n.d.*	0,150	0,087	5578	*n.d.*	0,32	0,32	4101,98
Sr	*n.d.*	0,00032	0,00011	6,33	3,95	*n.d.*	0,00088	10,58
Ba	0,02400	0,00067	0,00025	1,68	3,85	0,0670	0,0010	4,91
Ti	*n.d.*	*n.d.*	*n.d.*	316	52,15	*n.d.*	*n.d.*	864,79
V	0,630	0,0011	0,0011	17,5	1,20	0,310	0,0018	24,04
Cr	*n.d.*	0,0009	0,0018	50,3	16,95	*n.d.*	0,0053	11,57
Mn	0,083	0,017	0,0032	77,3	54,30	0,0580	0,046	148,11
Fe	0.00080	0,14	0,30	4815	675,55	0,00158	0,91	8616,83
Co	*n.d.*	0,00040	0,0013	4,11	0,85	*n.d.*	0,00070	4,98
Ni	0,0360	0,00048	0,0069	18,8	0,80	0,0350	0,011	8,80
Cu	*n.d.*	0,00049	0,0027	11,8	1,60	*n.d.*	0,0059	12,83
Zn	*n.d.*	0,085	0,048	4,31	2,40	0,232	0,65	13,35
Zr	*n.d.*	*n.d.*	*n.d.*	2,28	4,80	*n.d.*	*n.d.*	7,25
Nb	*n.d.*	*n.d.*	*n.d.*	0,16	0,30	*n.d.*	*n.d.*	0,90
Mo	0,230	0,0029	0,0045	0,020	0,50	0,230	0,0045	0,35
Ag	*n.d.*	0,00093	0,00083	0,0027	*n.d.*	*n.d.*	0,0009	*n.d.*
Cd	0,00000051	0,00013	0,00040	0,0056	0,00	*n.d.*	0,0021	*n.d.*
Hf	*n.d.*	*n.d.*	*n.d.*	0,057	0,20	*n.d.*	*n.d.*	0,18
Ta	*n.d.*	*n.d.*	*n.d.*	0,054	0,05	*n.d.*	*n.d.*	0,02
W	*n.d.*	*n.d.*	*n.d.*	0,74	0,30	*n.d.*	*n.d.*	0,00
Re	*n.d.*	*n.d.*	*n.d.*	*n.d.*	*n.d.*	*n.d.*	*n.d.*	0,0000
Hg	0,0000031	*n.d.*	*n.d.*	0,0024	*n.d.*	*n.d.*	*n.d.*	*n.d.*
Al	229,00	0,023	0,040	4426	1503,00	163,00	0,067	5010,47
Ga	*n.d.*	*n.d.*	*n.d.*	0,47	0,65	*n.d.*	*n.d.*	3,04
Ge	*n.d.*	*n.d.*	*n.d.*	*n.d.*	*n.d.*	*n.d.*	*n.d.*	0,24
In	*n.d.*	*n.d.*	*n.d.*	0,0037	*0,00*	*n.d.*	*n.d.*	0,01
Sn	*n.d.*	*n.d.*	*n.d.*	0,033	0,10	*n.d.*	*n.d.*	0,05
Sb	0,00000040	0,0038	0,0021	0,00093	0,00	*n.d.*	0,0017	0,02
Tl	0,00000031	0,0000057	0,0000077	0,00039	*n.d.*	*n.d.*	0,0000050	0,01
Pb	*n.d.*	0,00010	0,00013	0,59	*0,05*	0,00320	0,00047	0,20

Bi	0,00000090	*n.d.*	*n.d.*	0,0046	*n.d.*	*n.d.*	*n.d.*	0,00
B	*n.d.*	*n.d.*	*n.d.*	*n.d.*	*n.d.*	96,00	*n.d.*	*n.d.*
Si	24,00	*n.d.*	*n.d.*	10852	1386,20	17,00	*n.d.*	20281,70
As	0,510	0,0018	0,0018	0,0084	0,05	1,32	0,0020	0,02
Se	*n.d.*	0,0092	0,080	0,020	0,150	*n.d.*	0,0036	*n.d.*
Te	*n.d.*	0,00054	0,0026	*n.d.*	*n.d.*	*n.d.*	0,0010	*n.d.*
Sc	*n.d.*	*n.d.*	*n.d.*	1,73	0,90	*n.d.*	*n.d.*	1,93
Y	*n.d.*	*n.d.*	*n.d.*	0,78	1,90	*n.d.*	*n.d.*	2,98
La	*n.d.*	*n.d.*	*n.d.*	0,11	*0,55*	*n.d.*	*n.d.*	0,40
Ce	*n.d.*	*n.d.*	*n.d.*	0,36	1,35	*n.d.*	*n.d.*	1,14
Pr	*n.d.*	*n.d.*	*n.d.*	0,056	0,20	*n.d.*	*n.d.*	0,76
Nd	*n.d.*	*n.d.*	*n.d.*	0,27	0,55	*n.d.*	*n.d.*	0,81
Sm	*n.d.*	*n.d.*	*n.d.*	0,089	*0,00*	*n.d.*	*n.d.*	0,32
Eu	*n.d.*	*n.d.*	*n.d.*	0,040	0,10	*n.d.*	*n.d.*	0,17
Gd	*n.d.*	*n.d.*	*n.d.*	0,12	0,20	*n.d.*	*n.d.*	0,55
Tb	*n.d.*	*n.d.*	*n.d.*	0,019	0,00	*n.d.*	*n.d.*	0,05
Dy	*n.d.*	*n.d.*	*n.d.*	0,15	0,10	*n.d.*	*n.d.*	0,36
Ho	*n.d.*	*n.d.*	*n.d.*	0,036	0,05	*n.d.*	*n.d.*	0,10
Er	*n.d.*	*n.d.*	*n.d.*	0,082	0,10	*n.d.*	*n.d.*	0,21
Tm	*n.d.*	*n.d.*	*n.d.*	0,012	0,05	*n.d.*	*n.d.*	0,05
Yb	*n.d.*	*n.d.*	*n.d.*	0,087	0,15	*n.d.*	*n.d.*	0,24
Lu	*n.d.*	*n.d.*	*n.d.*	0,014	0,00	*n.d.*	*n.d.*	0,02
Th	*n.d.*	*n.d.*	*n.d.*	0,0080	0,00	*n.d.*	*n.d.*	0,07
U	*n.d.*	*n.d.*	*n.d.*	0,0028	0,00	*n.d.*	*n.d.*	0,03

Supplementary Information References

[1] Brown, K.L., Simmons, S.F. (2003) Precious metals in high-temperature geothermal systems in New Zealand. Geothermics 32, 619–625.

[2] Simmons, S. F., Brown, K. L., and Tutulo, B., M., (2016) Hydrothermal transport of Ag, Au, Cu, Pb, Te, Zn, and other metals and metalloids in New Zealand geothermal systems: Spatial Patterns, Fluid-mineral Equilibria, and Implications for Epithermal Mineralization: Economic Geology, p. 589-618.

[3] Hauksson, T., and Gudmundsson, 2008: Krafla. Acid wells. Landsvirkjun, report, 17 pp.

[4] Garbe-Schönberg, D., & Müller, S. (2014). Nano-particulate pressed powder tablets for LA-ICP-MS. Journal of Analytical Atomic Spectrometry., 29(6), 990–1000. doi:10.1039/c4ja00007b

[5] Paton, C., Hellstrom, J., Paul, B., Woodhead, J., & Hergt, J. (2011). Iolite: Freeware for the visualisation and processing of mass spectrometric data. Journal of Analytical Atomic Spectrometry, 26(12), 2508–2518. http://doi.org/10.1039/c1ja10172b

[6] Peters, D., & Pettke, T. (2016). Evaluation of Major to Ultra Trace Element Bulk Rock Chemical Analysis of Nanoparticulate Pressed Powder Pellets by LA-ICP-MS. Geostandards and Geoanalytical Research, 41(1), 5–28. doi:10.1111/ggr.12125

[7] Jochum, K. P., Nohl, U., Herwig, K., Lammel, E., Stoll, B., & Hofmann, A. W. (2005). GeoReM: A new geochemical database for reference materials and isotopic standards. Geostandards and Geoanalytical Research, 29(3), 333–338. http://georem.mpch-mainz.gwdg.de/ accessed August 2019)

[8] Schilling and Kingsley (2017) Platinum-group elements (PGE), Re, Ni, Cu, Ag and Cd variations along the Reykjanes Ridge and Iceland South-West Neovolcanic Rift Zone, from 50°N to 65°N: Implications on sulfide bearing PGE mantle source heterogeneities and partial melting effects. https://www.researchgate.net/publication/319354915

[9] Forest, A., Kelley, K. A., & Schilling, J. G. (2017). Selenium, tellurium and sulfur variations in basalts along the Reykjanes Ridge and extension over Iceland, from 50°N to 65°N (pp. 1–49).
